# A model-based framework for air quality indices and population risk evaluation, with an application to the analysis of Scottish air quality data

**DOI:** 10.1111/rssc.12001

**Published:** 2013-03

**Authors:** Francesco Finazzi, E Marian Scott, Alessandro Fassò

**Affiliations:** University of BergamoItaly; University of GlasgowUK; University of BergamoItaly

**Keywords:** Air quality indices, Ambient exposure and risk, Multivariate space–time models, Unbalanced networks

## Abstract

The paper is devoted to the development of a statistical framework for air quality assessment at the country level and for the evaluation of the ambient population exposure and risk with respect to airborne pollutants. The framework is based on a multivariate space–time model and on aggregated indices defined at different levels of aggregation in space and time. The indices are evaluated, uncertainty included, by considering both the model outputs and the information on the population spatial distribution. The framework is applied to the analysis of air quality data for Scotland for 2009 referring to European and Scottish air quality legislation.

## 1. Introduction

European legislation on air quality (Directive 2008/50/EC) identifies the needs for improved monitoring and assessment of air quality, including ‘to provide information to the public’.

The aim of this paper is to provide a model-based statistical framework for air quality assessment and the evaluation of population exposure and risk in a national context and applied to Scotland. The air quality strategy for Scotland is based on the European Commission directives, so a further aim of this paper is to develop and use a national Scottish air quality index as a scientific tool and as a source of public information.

The framework is applied to observed air quality data for Scotland for 2009 to provide a retrospective analysis of air quality and its expected effect on population at the country level. Indeed, the ultimate role of air quality assessment should be, on the one hand, to evaluate whether any actions that are undertaken to improve air quality have been successful or not (see Scott (2007)) and, on the other, to provide information about population risk and exposure. In particular, the main focus is on the definition of air quality, exposure and risk indices and on their evaluation at different levels of spatial and temporal aggregation. Each index represents a different aspect of pollution. The role of the air quality index is to provide concise information about the air quality level *per se* without any reference to the population exposure. However, exposure and risk indices consider also anthropological information and, hence, identify dangerous situations for the population's health.

The problem of assessing air quality over large regions and that of deriving indices is complex in several respects. The main complication arises from the way that airborne pollutants are measured in the field. The high economic costs of installation and maintenance of monitoring networks usually prevent pollutants from being measured with a spatial resolution that is adequate to assess exposure and risk with homogeneous accuracy all over the region.

The problem becomes more prominent when unbalanced monitoring networks are considered, i.e. when not all the pollutants are measured at each monitoring station. In such a case, it is not always clear how to define aggregated indices for the whole region and how to evaluate their uncertainty. Moreover, it is not straightforward to compare across years when, in each year, the structure of the monitoring network (sites included) and the quantity of missing data differ.

The above-mentioned problems are addressed by adopting, as the basis of the statistical framework, the dynamic coregionalization model (DCM) that has been introduced by Fassò and Finazzi (2011) and which can handle both unbalanced monitoring networks and missing data automatically. The DCM is used to evaluate the space–time correlation of the pollutants and their cross-correlation whereas the model output is used to define the indices and their uncertainty. In particular, the model output, in terms of the estimated pollutant concentrations, and the information about population distribution are combined to derive the exposure and risk indices.

In this work, the focus is on so-called ambient exposure rather than on personal exposure. A review of ambient exposure estimation methods can be found in Jerret *et al.* (2005), though limited to the intraurban case. A good example of personal exposure estimation, in contrast, can be found in Zidek *et al.* (2007), though it would be impractical to extend the personal exposure approach from city-size to country-size regions.

On the contrary, we aim to provide high resolution ambient exposure maps at the country level. Aware that this may introduce an ecological bias (according to Freedman (2001), ‘The ecological fallacy consists in thinking that relationships observed for groups necessarily hold for individuals’), we point out that our approach is an improvement with respect to the current air quality legislation which is based only on temporal averages of the measured pollutant concentration at the monitoring stations.

The rest of the paper is organized as follows. Section 2 is dedicated to the DCM. Parameter estimation and space–time pollutant concentration mapping are discussed for multivariate data observed in a heterotopic configuration. In Section 3, the problem of defining aggregated air quality indices for state-size regions is introduced and a model derived from the DCM is considered. Exposure and risk assessment indices based on coupling population spatial distribution and model outputs are defined in Section 4. Air quality data for 2009 that were collected over Scotland are considered in Section 5 and analysed within the statistical framework that is developed in this work.

## 2. The dynamic coregionalization model

Hierarchical models represent a useful statistical approach for the analysis of environmental data and they have been applied profitably in both frequentist and Bayesian contexts. Examples can be found in Banerjee *et al.* (2004) and Cressie and Wikle (2011) for the univariate and multivariate case. A comparison of different space–time hierarchical models in terms of prediction error is provided by Cameletti *et al.* (2011) though the comparison is limited to a particular data set. Since no general optimality results are available, we opt for the DCM which is flexible and easy to interpret. Moreover, as detailed in Finazzi and Fassò (2012), a software implementation of the DCM is readily available to download from http://code.google.com/p/d-stem/.

The DCM is a hierarchical multivariate space–time model based on latent variables which can handle both heterotopic data (non-colocated data) and missing data in a natural way. As a consequence, model estimation is based on the original data set as it has been acquired in the field without the need for any preliminary interpolation or missing data imputation.

Let 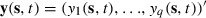 be the *q*-dimensional data response vector at the spatial location 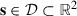 and at time 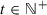. The general form of the model is


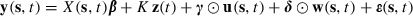
(1)

where *X*(**s**,*t*) is a matrix of known covariates, 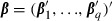 is a vector of global coefficients and ‘⊙’ is the Hadamard product. The *p*-dimensional latent temporal state 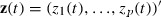 has the Markovian dynamics


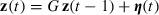
(2)

with *G* a stable transition matrix and Gaussian innovation ***η***∼*N*(0,Σ). The *q*×*p* matrix *K* is the loading matrix of known coefficients. The latent spatial component is modelled by both 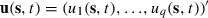 and 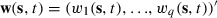 which are independent and identically distributed over time. For each fixed *t*, *u*_*i*_(**s**,*t*), 1≤*i*≤*q*, are independent latent zero-mean and unit variance Gaussian processes with spatial correlation function 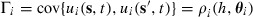, where *ρ*_*i*_ is a valid correlation function parameterized by ***θ***_*i*_ and *h*=‖**s**−**s**^′^‖ is the Euclidean distance between *s* and *s*^′^. In contrast, **w**(**s**,*t*) is described by a *q*-dimensional linear coregionalization model (LCM) of *c* components


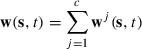
(3)

where **w**^*j*^(**s**,*t*), 1≤*j*≤*c*, are independent latent zero-mean and unit variance Gaussian processes with correlation matrix function 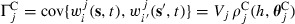, 1≤*i*,*i*′≤*q*, 1≤*j*≤*c*. Each *V*_*j*_ is a correlation matrix and each 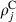 is, again, a valid correlation function (see Wackernagel (2003) for an introduction to the LCM).

The vectors 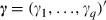 and 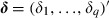 contain the scale parameters. Finally, 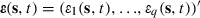 is the measurement error which is assumed to be white noise in space and time. In particular, 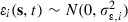, 1≤*i*≤*q*. The parameter set to be estimated is


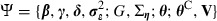
(4)

where 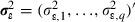, 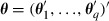, 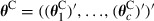 and 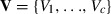.

The DCM defined in equation (1) is flexible in the sense that the covariates *X*(**s**,*t*) and the latent variables **z**(*t*), **u**(**s**,*t*) and **w**(**s**,*t*) describe different aspects of the phenomenon under study and they can be included and excluded from the model depending on what is the focus of the data analysis. The covariates (if available) and the latent spatial variables **u**(**s**,*t*) and **w**(**s**,*t*) are to be included when the model is used for mapping whereas the latent temporal variable **z**(*t*) should be included when the focus is on the temporal dynamics.

The main difference between **u**(**s**,*t*) and **w**(**s**,*t*) is that each *u*_*i*_(**s**,*t*), 1≤*i*≤*q*, is characterized by its own correlation function 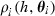 whereas the LCM imposes, for each of the *c* components, a unique correlation function across variables. It can be said that **u**(**s**,*t*) is the direct component whereas **w**(**s**,*t*) is the interaction component. Although the model can be estimated with both the components included, preliminary studies suggest that, when real data are considered, it is important to choose one component or the other depending on the spatial correlation structure of the data. Indeed, the LCM should be included solely when data are known to be spatially cross-correlated whereas the direct component should be considered solely when the correlation functions describing the *q*-variables are expected to be different. In this latter case, it is still worthwhile to consider the model in its multivariate form since the *q*-variables may be temporally cross-correlated. Finally, if the flexibility of model (1) must be increased, a version of the DCM with spatiotemporal varying coefficients can be considered as detailed in Finazzi and Fassò (2011).

The matrix 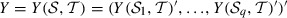 is the *N*×*T* matrix of all the observations collected at locations 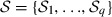 and time 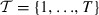. Here, 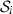 is the collection of *n*_*i*_ locations where the variable *y*_*i*_ is observed and 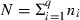. The *n*_*i*_ are not constrained to be equal; nor are the 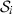. Thus, the fully heterotopic case is admitted, i.e. the variables can be observed over disjoint sets 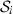 of spatial locations. This includes the above-mentioned case of unbalanced monitoring networks. Also note that the matrix *Y* may include missing data.

The maximum likelihood estimate of Ψ is obtained by the expectation–maximization (EM) algorithm as described in Fassò and Finazzi (2011). The whole estimation procedure has been proven to be stable even when large data sets or large parameter sets are considered. This is largely due to the quasi-closed form property of the EM steps for this model. Note that the standard deviations of the estimated model parameters are directly obtained from an approximated Fisher information matrix 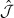 computed as in Fassò*et al.* (2009).

Let 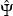 be the maximum likelihood estimate of Ψ; then the concentration of the *i*th pollutant at a new set of sites 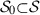 and time 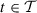 is evaluated by means of a plug-in approach as


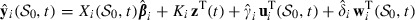
(5)

where 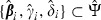, 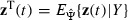 is the Kalman smoother output, 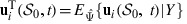 and 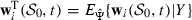 are the estimated latent spatial variables, 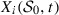 is the matrix of covariates and *K*_*i*_ is again the loading matrix. Note that the Kalman smoother provides a fast algorithm for the evaluation of 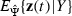 using the state space representation of model (1) (see for instance Shumway and Stoffer (2006)). In contrast, the conditional expectations of the latent spatial variables with respect to the observed data are evaluated through the usual formulae of the multivariate normal distribution adapted for the missing data case as detailed in Fassò*et al.* (2009).

The spatial prediction variance–covariance matrix of 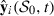 is given by


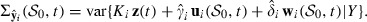
(6)

If the sites in 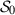 cover the whole region 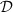 as a fine regular grid, we call 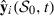 a map and the ordered collection


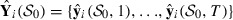
(7)

a dynamic map for the *i*th pollutant. If, instead of the set of sites 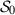, a tessellation 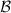 of the region 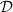 is considered, the change-of-support problem (see Gotway and Young (2002)) must be addressed and


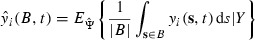
(8)

must be evaluated for each block 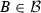. However, if the blocks in 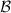 are not too large with respect to the spatial variability scale of *y*_*i*_(**s**,*t*), then 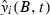 can be replaced by 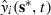, with s^*^ the centre of the pixel *B*. In what follows, the dependence on the estimated parameter set 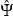 will be dropped to simplify the notation.

It should be noted that the dynamic map carries all the information about the temporal and the spatial dynamics of the ground level pollutant concentration. However, the amount of information is huge and it is rarely useful to decision makers. The following sections describe how aggregate information (uncertainty included) can be derived by taking advantage of the flexibility of the DCM and by considering both the estimated model latent variables and the estimated pollutant concentrations.

## 3. Global air quality indices

When environmental space–time data are considered, aggregation is often useful over either space or time. Obtaining aggregated results over time is usually straightforward as the data are usually collected at regular time steps. Spatial aggregation is more complex as the spatial sampling locations are irregularly sparse over the region. For these reasons, the focus of this section is on aggregation over space; in particular, the problem of defining global air quality indices with measures of uncertainty is addressed.

With a global air quality index, we refer here to a single number that can describe, for a region 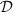 at time *t*, the air quality in terms of the monitored pollutants. Hence, a global air quality index represents, on the one hand, a simple and concise reporting measure for the public and, on the other, a measure to compare different temporal periods with respect to air quality easily.

From a statistical point of view, the global air quality index is considered here as a latent variable which manifests itself through the pollutants’ concentration measurements collected at the sampling sites. Although in a different context, the same idea has been developed by Chiu *et al.* (2011) in the definition of health factor indices.

To define the latent global air quality index, the following multivariate model is proposed:


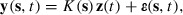
(9)

which is a reduced version of model (1). This kind of model has been used in various environmental applications. For example, for large data sets, using the so-called fixed rank smoothing approach of Cressie and Johannesson (2008), the matrix *K*(**s**) is defined by a set of fixed spatial basis functions. In ecological trend analysis, Zuur *et al.* (2007) used the so-called dynamic factor model to estimate the common trend of a non-large number of time series. To do this, the matrix *K*(**s**) is estimated by using suitable constraints.

In our case, the global air quality index should represent a common trend at the monitoring stations but the number of times series can be high and the matrix *K*(**s**) is poorly identifiable. Hence, we avoid identifiability problems by fixing *K*(**s**) to agree with the station averages. The dimensionality of **z**(*t*) is equal to 1 or to the total number of pollutants *q*. The first case may be considered when the pollutants are highly positively correlated. The **z**(*t*) is hence unidimensional and the global air quality index can be defined as


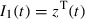
(10)

where *z*^T^(*t*) is the estimated latent state output of the Kalman smoother. In the second case, when the pollutants are not positively correlated, it is better to rely on a *q*-variate **z**(*t*) and each pollutant retains its own temporal trend. Following the aggregation approaches of Bruno and Cocchi (2002) and Lee *et al.* (2011), two possible global air quality indices are


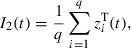
(11)


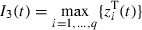
(12)

where 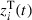 is the *i*th component of the estimated latent state **z**^T^(*t*) output of the Kalman smoother.

With regard to the uncertainty that is related to the above indices, this can be evaluated by considering the variance–covariance matrix *P*^T^(*t*) related to **z**(*t*). In particular, the variance of *I*_2_(*t*) can be evaluated as


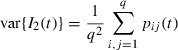


where *p*_*ij*_(*t*) is the (*i*,*j*)th element of the matrix *P*^T^(*t*). From 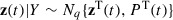, a 95% confidence interval for *I*_2_(*t*) can be evaluated as 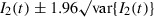. Confidence intervals for *I*_3_(*t*) do not have, in general, a simple closed form but they can be easily evaluated by considering the quantiles of 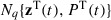.

When plotted against time, the indices *I*_1_, *I*_2_ and *I*_3_ provide an immediate view of the air quality trend over the region considered. This allows comparison across days and years and to test whether air quality is either improving or worsening over time. From an epidemiological point of view, however, this kind of information is not sufficiently rich to derive conclusions about the potential effect of pollution on population health, which is the object of the next section.

## 4. Population exposure and risk assessment

Mapping pollutant concentration over space and time is important to identify critical areas with respect to air quality. To evaluate the potential effect of airborne pollution on population health, however, the spatial distribution of the population must also be considered. Hence, population exposure and population risk are evaluated by analysing the interaction between the spatial distributions of the pollutants and population.

### 4.1. Exposure index

Exposure and risk are related concepts and the respective indices may carry similar information. However, in particular contexts, exposure and risk might differ substantially. In a way, the risk index should be related to the chance of extreme events whereas the exposure index should be related to the number of people who are exposed to a given pollution level.

The exposure index for the *i*th pollutant, the block 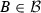 and the temporal frame 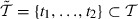 are defined here by


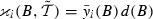
(13)

where


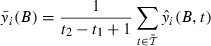


is the estimated temporal average concentration of the *i*th pollutant and *d*(*B*) is the (time invariant) population count of block *B*. In this case, we prefer to evaluate a temporally averaged index since, as said before, the exposure index should reflect the long-term effect. For instance, the set 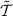 can represent a month, a whole season or a year. If needed, the exposure index 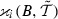 can be aggregated over space to define the following average exposure index for region 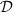:


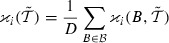
(14)

where *D* is the total population count of region 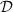.

To evaluate how the spatial distributions of population and pollutant concentration interact, an interesting picture is provided by the cumulative exposure distribution, which is given by


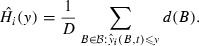
(15)

### 4.2. Risk index

The risk index is defined here by considering a concentration threshold *L* for a pollutant above which the effect on human health is known to be significant. The threshold *L* can be, for example, the concentration level which causes respiratory hospital admissions to increase with respect to a baseline rate. Given this threshold, two central quantities are the probability that the pollutant concentration *y*_*i*_ exceeds the *L*-threshold in block *B* and time *t*, namely


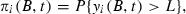
(16)

and the probability that the number of days for which *L* is exceeded in block *B* exceeds *M*, i.e.


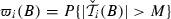
(17)

with 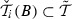 the set of days for which the exceedance occurs.

The following risk indices are considered:


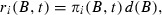
(18)


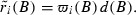
(19)

In particular, the risk index that is defined in equation (19) reflects the current air quality norm, which usually prescribes a maximum number of days that the pollutant concentration can exceed a threshold *L*. The risk index of equation (19) can be evaluated by noting that


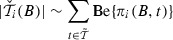
(20)

with Be(*p*) the Bernoulli random variable with parameter *p*. Since the sum of independent Bernoulli random variables with varying parameter *p* has no simple closed formula, expression (20) can be evaluated numerically. For instance, the distribution of the number of days 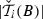 can be evaluated by Monte Carlo simulation. In practice, the probabilities in equations (18) and (19) are computed by using the estimated parameter set 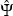.

As a final remark, it is worth noting that the exposure and risk indices that were defined above are conditioned on the observed covariates and the estimated latent variables **u**, **w** and **z**. In other words, both indices must be applied only in retrospective analysis and they cannot be considered as characteristics of a particular spatial site 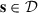 independent of time.

### 4.3. Exceedance probability evaluation

A key aspect in assessing risk is the evaluation of the exceedance probability 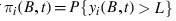. Although, for fixed 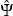, 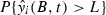 can be easily evaluated, it gives a conservative estimate of *π*_*i*_(*B*,*t*) as it does not take into account any model misspecification error and 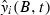 is smoother than the real pollutant concentration *y*_*i*_(*B*,*t*). Moreover, we are interested in evaluating confidence intervals for *π*_*i*_(*B*,*t*), which are not provided by the dynamic kriging. For these reasons, the following procedure is considered.

- The leave one site out cross-validation technique is applied and the cross-validation residuals *e*(**s**,*t*), **s** ∈ *S*_*i*_, are considered. In particular, *e*(**s**,*t*) is computed by using data **Y**_−**s**_ which is the data matrix omitting all the data from site **s**.
- Residuals are Studentized with respect to the dynamic kriging variance 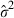, namely
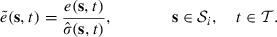
(21)
- Considering all the Studentized residuals 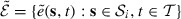, their cumulative distribution function 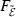 is obtained by kernel smoothing.
- For each block *B* and time *t*, the exceedance probability is evaluated as
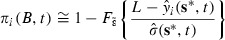
(22)
  with 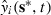 the kriged pollutant concentration under the estimated model with parameter set 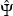.

The cross-validation residuals are presumed to take into account the model misspecification error and they are characterized by a higher variance with respect to classical residuals. The transformation at step (b) of this procedure is not a real Studentization (Cook, 1982) since *σ*^2^(**s**,*t*) is not an estimate of the residual standard deviation 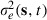. However, 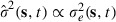 and the Studentization procedure is applied here to homogenize the model residuals which, on their own, are not homoscedastic with respect to space. Indeed, the cumulative distribution function 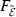 can be evaluated by considering all the Studentized residuals provided that they are white noise in both space and time. Finally, in step (d) the approximations 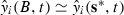 and
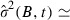

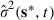
 are considered negligible since it is assumed that 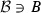 is a fine tessellation of the region 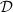.

To evaluate whether the probabilities given by expression (22) are reliable, we provide confidence intervals as follows. We sample from the asymptotic distribution of the estimated parameter set, 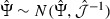, where 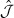 is the approximated Fisher information matrix of Section 2. As a consequence, we obtain a collection of *R* parameter sets 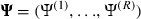. For each Ψ^(*j*)^ we evaluate bootstrap replications of 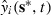 and 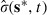 and we compute *π*^(*j*)^(*B*,*t*) by using equation (22). The confidence intervals are based on the sample quantiles of 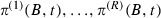.

## 5. Analysis of the Scottish air quality data for year 2009

The methodology that was discussed in the previous sections is applied here to Scottish air quality data for the year 2009. The aims are to obtain better insight into the spatiotemporal dynamics of the principal airborne pollutants, to evaluate population exposure and risk and to understand whether the air quality monitoring network is appropriate to answer the above questions or whether it should be strengthened in terms of number of monitoring stations and spatial distribution. The air pollution standards and the air quality objectives that are considered in the analysis are based on the ‘Air quality standards (Scotland) regulations 2007 for the purpose of local air quality management’. A summary of the current standards and objectives can be found in Department for Environment, Food and Rural Affairs (2009).

This section is organized as follows. Section 5.1 describes the data considered in terms of pollutants, population distribution and covariates. The global air quality index for Scotland is evaluated in Section 5.2 whereas population exposure and risk indices are developed in Section 5.3.

### 5.1. Description of the data

The sources of data that are considered in this work are essentially three: the ground level concentration of airborne pollutants, measured by the Scottish automatic urban network, the population spatial distribution downloaded from the Oak Ridge National Laboratory and the meteorological covariates downloaded from the Nasa Global Modeling and Assimilation Office. Each source of data is characterized by a different spatial and temporal resolution, as described hereafter.

#### 5.1.1. Pollutant concentrations

The Scottish automatic urban network provides hourly mean data on six main airborne pollutants, namely nitrogen dioxide (NO_2_), ozone (O_3_), carbon monoxide (CO), sulphur dioxide (SO_2_) and particulate matters PM_10_ and PM_2.5_. In this work, only the NO_2_, O_3_ and PM_10_ concentration data are considered since they are measured at sufficient monitoring stations to justify a space–time analysis. Moreover, the hourly mean data are averaged to work with daily data.

**Fig. 1:**
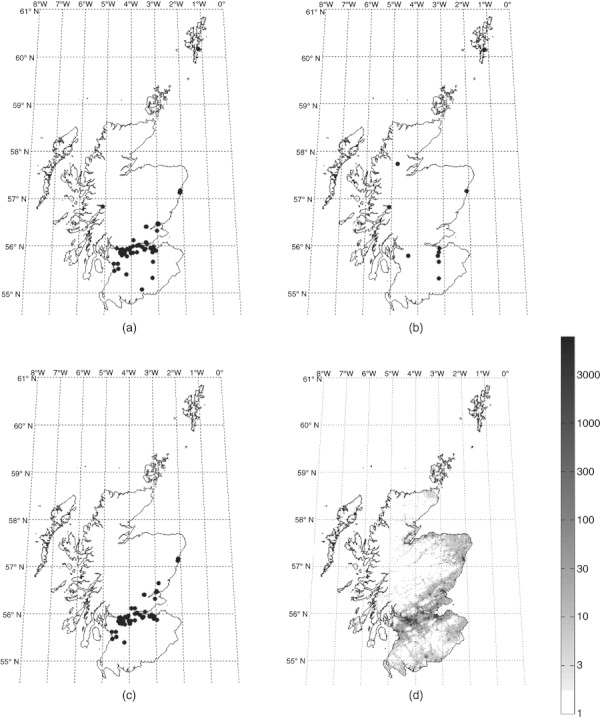
Spatial distributions of the Scottish automatic urban network monitoring sites: (a) NO_2_ (66 sites); (b) O_3_ (10 sites); (c) PM_10_ (60 sites); (d) population spatial distribution

For the year 2009, the number of monitoring stations is 81. Each station measures only a subset of the three pollutants considered and missing data are possible, due to temporary breakdowns of either the station or the single measuring instrument. Days with less than 75% hourly data (18 h) are considered as days with missing data. The exact number of monitoring stations for each pollutant is reported in Table 1, showing that the network is unbalanced in the sense of Bodnar *et al.* (2008). The respective spatial distributions are reported in Figs 1(a)–1(c), from which it is clear that the monitoring stations are not evenly distributed over Scotland as they are mainly in the most populated areas.

**Table 1:** Summary statistics of the pollutant concentration data for 2009

*Pollutant*	*Number of stations*	*Mean* (*μgm*^−3^)	*Standard deviation* (*μgm*^−3^)	*Missing (%)*
NO_2_	66	32.19	23.35	12.7
O_3_	10	55.80	18.99	12.1
PM_10_	60	16.60	8.58	16.1

#### 5.1.2. Population distribution

The population distribution has a twofold role here. It is considered as a time invariant covariate and it is used to evaluate the exposure and risk indices that were discussed in Section 4. The Oak Ridge National Laboratory manages the LandScan™ ambient population count database, currently updated to the year 2008 (see Bhaduri *et al.* (2007)). The database provides estimates of 24-h average population counts over the entire world with 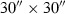 resolution (approximately 1 km×1 km). The population spatial distribution for Scotland is reported in Fig. 1(d), from which it is clear that most of the population is in the central belt along the Glasgow–Edinburgh parallel.

#### 5.1.3. Morphological and meteorological covariates

Pollutant concentrations are known to be related to some anthropological and meteorological covariates owing to the physical processes that drive the pollutant diffusion and advection. In this work, we consider population count pop, sea level pressure slp, temperature *t*, specific humidity sh, wind speed ws and boundary layer height blh. In particular, the boundary layer height is the height of the lowest part of the troposphere that is directly influenced by the ground and it determines the volume that is available for pollutants to disperse. Note that the population count is a good proxy of both the pollutant emissions and the site type (urban, suburban and rural). The meteorological covariates are downloaded from the Nasa Global Modeling and Assimilation Office. In particular, the ‘Modern era retrospective analysis for research and applications’ product (see Rienecker *et al.* (2011)) is considered, which is characterized by a temporal resolution of 1 h and a spatial resolution of 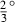 longitude by 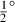 latitude. Since the pollutant concentrations are daily averages, the meteorological covariates are also averaged over 24 h and they are interpolated at 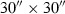 resolution for mapping purposes.

As a final remark we point out that, though the concentration data might be preferentially sampled (see Diggle *et al.* (2010)), the problem is largely mitigated by the covariates and in particular by the population distribution which acts as a proxy for the pollutant emissions. Net of the covariates, the ‘residual’ data 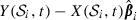 can be assumed to be not preferentially sampled with respect to the residual random field 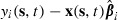, ∀*i*,*t*. Moreover, since the monitoring stations are not very high in number, we believe that the population distribution, which is available at high spatial resolution, is more informative than the network itself on the preferential sampling.

### 5.2. Global air quality index estimation

The methodology that was discussed in Section 3 is here considered to evaluate a global air quality index for Scotland for 2009. Since NO_2_, O_3_ and PM_10_ are known to have different temporal dynamics over the year, the global air quality index (12) is considered. With regard to the estimation of the latent temporal state **z**(*t*), model (9) is considered with


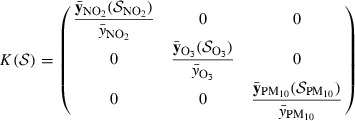
(23)

where


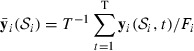


is the temporal average scaled pollutant concentration at the sampling sites 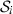 for the *i*th pollutant, 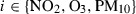 whereas 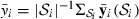 is the network average scaled pollutant concentration. The scaling factors *F*_*i*_ have a different role from that of the concentration thresh olds *L*. The latter are usually provided by the air quality legislation as thresholds that should not be exceeded whereas the former are used to homogenize the pollutants in the case that they are provided by using different statistics (average, running average, maximum etc.).

The UK air quality index and banding system (IBS) approved by the UK Committee on Medical Effects of Air Pollution Episodes is characterized by a 1–10-index divided into four bands, namely low, moderate, high and very high. Each index value corresponds to a range of concentration where the pollutant concentration can fall when measured over a period of time Δ*T*. The limits of each range depend on the particular pollutant as well as Δ*T*. For PM_10_, an index value equal to 10 corresponds to a running 24-h mean concentration 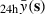 equal to or higher than 128 *μ*g m^−3^. Although the data that are considered in this work are daily average concentrations rather than running 24-h means, it makes sense to consider 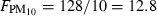. The division by 10 is introduced to keep the index *I*_3_ comparable with respect to the index of the IBS. The scaling factors for NO_2_ and O_3_ are not immediately available since the IBS prescribes ranges for the running 8-h mean 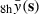 for O_3_ and the hourly mean 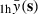 for NO_2_. Preliminary analysis not reported here suggests that


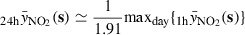


and


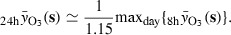


The scaling factors are then chosen to be 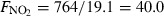 and 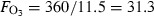, where 764*μ*g m^−3^ and 360*μ*g m^−3^ are the concentrations corresponding to an index value equal to 10 in the IBS for NO_2_ and O_3_ respectively.

**Fig. 2:**
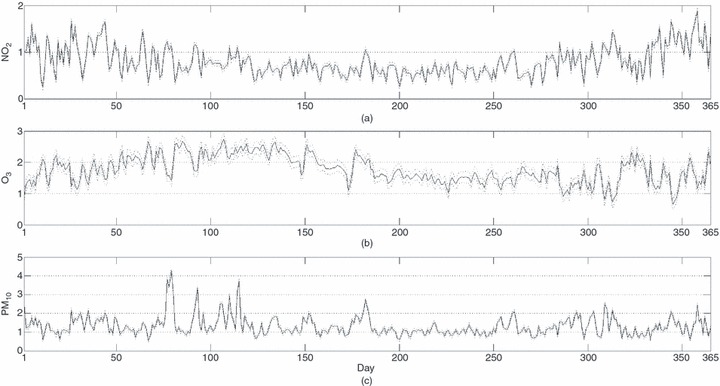
Kalman smoother output **z**^T^(*t*) of model (9) (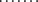, error bounds 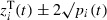): (a) NO_2_ component; (b) O_3_ component; (c) PM_10_ component

**Fig. 3:**
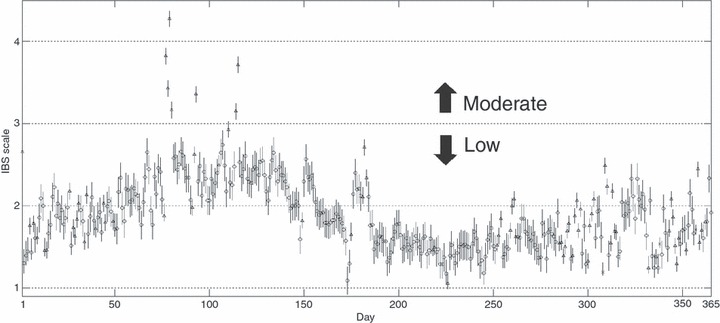
Scotland daily air quality assessment through the air quality index *I*_3_ for the year 2009 (the pollutant that gives rise to the maximum indicated by the marker): |, 95% confidence interval; x, NO_2_; O, O_3_; Δ, PM_10_

Using the resulting loading matrix 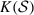, model (9) is estimated by the EM algorithm and the related temporal component **z**^T^(*t*) is depicted in Fig. 2, where error bounds are defined as 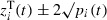, with *p*_*i*_(*t*) the *i*th diagonal element of var{**z**(*t*)|*Y*}. Note that each pollutant is characterized by a different temporal dynamic as expected. O_3_ peaks in March or April whereas NO_2_ peaks in winter. The particulate matter PM_10_ does not show a clear trend and the peaks are a consequence of unfavourable meteorological conditions. Fig. 3 shows the evaluated air quality index *I*_3_(*t*) which is representative of Scotland as a whole. By analysing the temporal series of *I*_3_(*t*), it can be concluded that, during the year 2009, air pollution over Scotland remained low with the exception of three events spread over 7 days during March and April. All the events can be associated with moderate concentration levels of PM_10_ due to adverse meteorological conditions. Note, moreover, that the decisive pollutants are O_3_ and PM_10_ whereas NO_2_ is identified in Fig. 3 only three times.

### 5.3. Population exposure and risk evaluation

As discussed in Section 4, the evaluation of population exposure and risk is based on coupling population spatial distribution and estimated pollutant concentrations obtained as output of the DCM. The DCM defined in equation (1) allows joint modelling of the space–time correlation of all the pollutants considered. However, to define the parametric structure of the multivariate model better, it is useful first to estimate one univariate model for each pollutant. In fact, the dimension *p* of the latent temporal state **z**(*t*), the inclusion of either **u**(**s**,*t*) or **w**(**s**,*t*) (or both) and the number *c* of coregionalization components must be decided before estimating the model. Although multivariate models can be compared by means of cross-validation techniques, the number of possible model parameterizations may be large and it is useful to consider the univariate models as a guide to define the parameterization of the multivariate model. As far as the spatial correlation structures is concerned, the exponential correlation function has been considered, namely 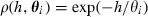, 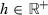, 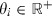.

Table 2 reports the value of 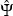 computed by means of the EM algorithm for each of the univariate models. All the variables and the covariates have been log-transformed and standardized. Standardization helps numerical stability and allows a direct comparison of the parameter values across pollutants.

**Table 2:** Estimated parameters for the univariate DCMs and respective cross validation mean-squared error cmse

*Pollutant*	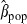	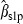	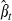	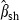	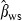	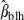
NO_2_	0.464	−0.040	0.321	−0.473	−0.197	−0.220
Standard deviation	0.005	0.021	0.065	0.055	0.015	0.017
O_3_	−0.090	−0.229	0.392	−0.297	0.216	0.166
Standard deviation	0.016	0.026	0.082	0.069	0.018	0.021
PM_10_	0.121	0.224	0.289	−0.294	−0.084	−0.222
Standard deviation	0.006	0.038	0.078	0.068	0.020	0.021

	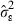	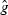	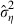	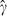	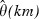	*cmse*
NO_2_	0.367	0.901	0.010	0.463	62.30	0.483
Standard deviation	0.003	0.038	0.001	0.010	4.28	
O_3_	0.274	0.951	0.027	0.427	136.47	0.561
Standard deviation	0.014	0.019	0.002	0.020	21.96	
PM_10_	0.286	0.674	0.137	0.516	88.36	0.366
Standard deviation	0.002	0.054	0.019	0.017	8.91	

By comparing the values of the estimated 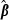-parameters with respect to their standard deviations, it can be seen that all the covariates are significant except 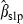 for NO_2_. The estimated parameters 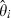 are expressed in kilometres and they describe the strength of the spatial correl ation of the latent variables *u*_*i*_(**s**,*t*). In particular, in the case of the exponential correlation function, the spatial correlation between any two points in space is around 0.05 when their distance is 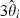. Note that 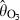 has the highest value but also the highest standard deviation. This is because the O_3_ monitoring network is very sparse and cannot capture a possible high spatial frequency of 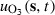. This consideration suggests that an LCM may be appropriate for modelling a spatial latent component that is common to all the pollutants, even if there is not enough evidence to conclude that the 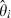 are equal. In contrast, the 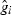-values are significantly different, suggesting that each pollutant should retain its own temporal dynamics; hence *p*=3 for the multivariate model. The optimal number *c* of coregionalization components has been assessed through cross-validation, suggesting *c*=1. The cross-validation mean-squared errors cmse that are obtained by applying the leave one site out technique are reported in Table 2.

Considering now the multivariate model (1), the estimated 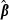, 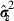 and 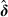 are reported in Table 3 whereas the remaining parameters are


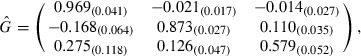
(24)


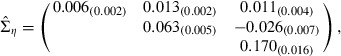
(25)


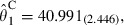
(26)


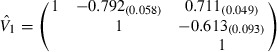
(27)

with the standard deviations in parentheses. The 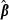-coefficients related to the covariates are all significantly different from 0 and their sign is in line with the physics of the pollution phenomenon. For instance, as expected, 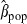 is positive for NO_2_ and PM_10_ whereas it is negative for O_3_, implying, on average, a lower concentration of O_3_ in low populated areas. The analysis of the matrix 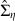, and in particular the variances on its diagonal, suggests a low contribution of the latent temporal variable **z**(*t*) in explaining the NO_2_ and O_3_ variability whereas the contribution is more relevant for PM_10_. In contrast, the analysis of 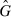 reveals the slow temporal dynamics of NO_2_ and O_3_ (diagonal elements close to 1) which can be related to the temporal persistence of the pollutants not accounted for by the covariates. The parameter 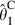 of the spatial correlation function is common for all the pollutants considered and it is also expressed in kilometres. The matrix 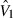 shows that the component of **w**(**s**,*t*) that is related to O_3_ is negatively correlated with the components that are related to the other pollutants. Note that 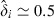 for each pollutant, namely **w**(**s**,*t*) accounts for about half of the data variability. By comparing the cmse-values reported in Tables 2 and 3, the gain in terms of prediction capability for the multivariate model can be appreciated. The reduction in cmse is particularly evident for O_3_ which has the spar sest monitoring network and benefits more from the spatiotemporal correlation with the other pollutants. Note that this is a major benefit of using a multivariate model and in particular the DCM.

**Table 3:** Subset of the estimated parameters for the multivariate DCM

*Pollutant*	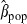	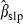	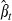	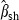	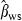	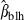	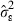	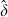	*cmse*
NO_2_	0.447		0.309	−0.415	−0.192	−0.211	0.317	0.567	0.439
Standard deviation	0.005		0.056	0.015	0.018	0.017	0.004	0.010	
O_3_	−0.166	−0.196	0.368	−0.251	0.208	0.188	0.243	0.451	0.478
Standard deviation	0.016	0.025	0.079	0.066	0.017	0.020	0.014	0.019	
PM_10_	0.089	0.266	0.382	−0.285	−0.064	−0.227	0.244	0.571	0.339
Standard deviation	0.005	0.034	0.077	0.068	0.020	0.022	0.003	0.011	

The estimated multivariate model is then used to evaluate population exposure and risk with respect to each pollutant. In particular, the dynamic maps 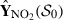, 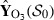 and 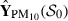 are evaluated over the regular grid 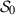 with spatial resolution 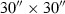 within the Scottish boundaries.

**Fig. 4:**
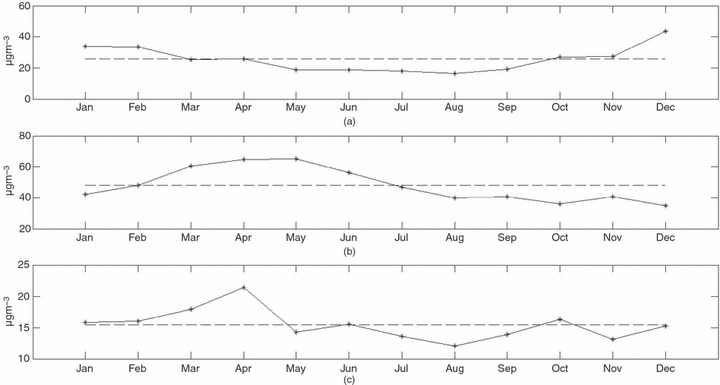
Monthly and yearly (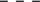) average population exposure: (a) NO_2_; (b) O_3_; (c) PM_10_

**Fig. 5:**
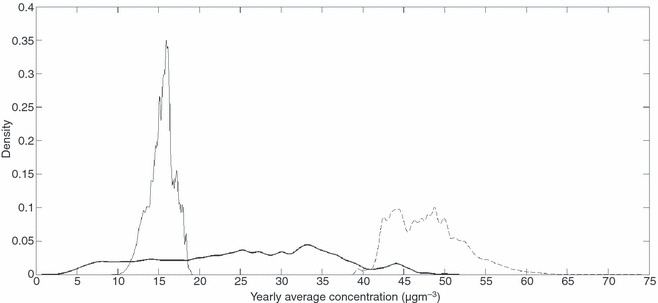
Yearly average exposure distribution: 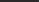, NO_2_; 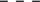, O_3_; 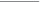, PM_10_

As a first result, Fig. 4 shows the monthly and the yearly average population exposure evaluated by considering the exposure index (14), which can be related to an average Scottish person. Moreover, Fig. 5 displays, for each pollutant, the yearly average exposure distribution based on the cumulative distribution (15) and evaluated by considering a Gaussian kernel smoother with bandwidth 0.5 *μ*g m^−3^. By looking at the results of Fig. 5, it can be noted that the exposure distributions differ greatly across pollutants. In particular, most of the Scottish people are exposed to the same yearly average PM_10_-concentration ±5 *μ*g m^−3^, whereas this is not true for NO_2_ which is more spread out. Moreover, the exposure distribution of O_3_ is characterized by a prominent right tail representing people living in rural areas, where the concentration of O_3_ is higher. Although the graphs are reported on the same axis, they are not directly comparable in terms of health effects.

**Fig. 6:**
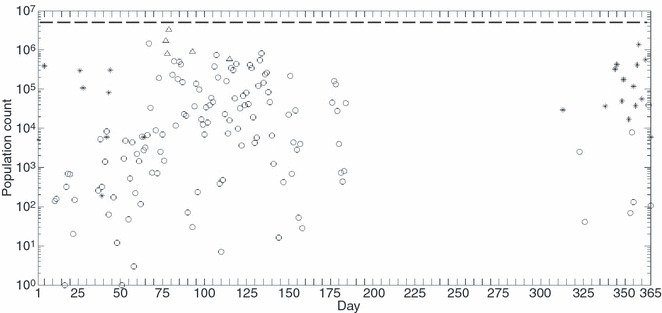
Daily exposure time series (number of people exposed to a pollutant concentration exceeding the threshold): 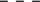, total population of Scotland; O, O_3_>87 *μ*g m^−3^; Δ, PM_10_>50 *μ*g m^−3^; 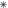, NO_2_>105 *μ*g m^−3^

The daily exposure time series given by 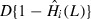 are reported in Fig. 6, where *D* is the total population count and 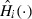 is given by equation (15). The thresholds *L* are 105, 87 and 50 *μ*g m^−3^ for NO_2_, O_3_ and PM_10_ respectively and they have been derived from the ‘Air quality standards (Scotland) regulations 2007’ following arguments similar to those of the previous paragraph. The time series disappear between day 190 and day 315 since the thresholds are never exceeded from July to October.

To compute the risk indices (18) and (19), the exceedance probabilities and their respective confidence intervals have been evaluated for each day and each pollutant at 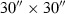 of resolution. In particular, the confidence intervals have been obtained by considering *R*=100 bootstrap replications as detailed in Section 4.3 and by analysing, for each map pixel and each day, the respective empirical cumulative distribution function.

**Fig. 7:**
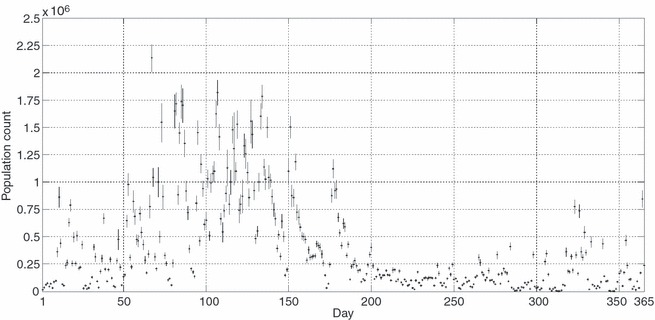
Aggregated O_3_ risk index and 95% confidence intervals (|) (with respect to *L*=87 *μ*g m^−3^)

The daily exceedance probabilities can be used for evaluating daily risk maps or aggregated risk indices. As an example, Fig. 7 shows the daily time series and the respective 95% confidence interval of the risk index for O_3_ and *L*=87 *μ*g m^−3^ aggregated at the country level.

**Fig. 8:**
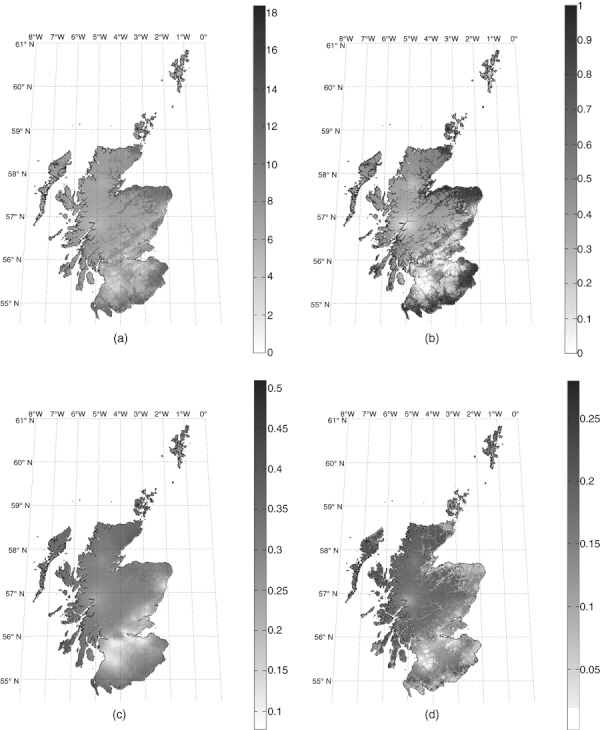
(a) Map of the estimated average number of days of exceedances for PM_10_, (b) map of the probability of exceedance of the 7-days limit, (c) standard deviation of map (a) and (d) range of the 95% confidence interval on map (b)

With regard to the number of days of exceedance, Fig. 8(a) reports the map of the average number of days of exceedance for PM_10_ whereas Fig. 8(b) reports the probability map that the threshold *L* has been exceeded for more than 7 days, which is required to evaluate the risk indicator that is defined in equation (19). Note that the 7-days limit represents one of the objectives of the Scotland national air quality strategy to be achieved by December 31st, 2010. Both the average number of days of exceedance and the probability of exceedance of the 7-days limit have been evaluated by considering the daily exceedance probability maps and by simulating from the distribution that is defined in equation (20). In particular, the results that are reported in Fig. 8(a) are based on 500 Monte Carlo simulation runs (see Section 4.2). The measures of uncertainty that are reported in Figs 8(c) and 8(d) have been obtained by repeating the same simulation approach for the *R*=100 bootstrap replications of daily exceedance probability maps.

The probability that the threshold of 50 *μ*g m^−3^ has been exceeded for more than 7 days is higher in the Grampian region (north-east) and along the southern border of Scotland rather than in cities such as Glasgow or Edinburgh. However, this is a consequence of the fact that those regions are poorly covered by monitoring stations and the uncertainty in the estimated concentration of pollutant is high. The north-west regions are not covered as well but they are characterized by a very low PM_10_-concentration and the exceedance probability is not so high despite the uncertainty. These last considerations suggest that the Scottish air quality network should be improved by installing additional monitoring stations in those areas which are uncovered and are characterized by a high risk and/or a high uncertainty. This is particularly true for O_3_ that is currently monitored at only 10 locations.

## 6. Conclusions

Assessing air quality at the country level and evaluating the related population exposure are challenging tasks. In this paper, both problems have been addressed by developing a statistical framework based on a flexible multivariate space–time model, the DCM, and on a set of aggregated indices built by considering both the model output and the information of the population spatial distribution.

The DCM is sufficiently flexible to accommodate the data complexity related to ground level unbalanced networks and it naturally copes with the inevitable missing data problem. The indices are accompanied by measures of uncertainty and they can be provided at different levels of temporal and spatial aggregation to study different aspects of the pollution phenomenon. The global air quality indices allow us to compare different time periods with respect to the air quality at the country level easily and readily. In contrast, the exposure and risk indices provide an effective way to identify critical areas with respect to air quality and useful information to improve the ground level monitoring network.

As a whole, the statistical framework has been proven able to assimilate the current air quality legislation, both at the European and at the national levels, and to provide easily interpretable results for decision makers. The statistical framework has been successfully applied to the analysis of the Scottish air quality data for the year 2009, shedding light on aspects related to population exposure and risk that have never been investigated before.
